# Analysis of digital competence of educators (DigCompEdu) in teacher trainees: the context of Melilla, Spain

**DOI:** 10.1007/s10758-021-09546-x

**Published:** 2021-07-14

**Authors:** José Manuel García-Vandewalle García, Marina García-Carmona, Juan Manuel Trujillo Torres, Pablo Moya Fernández

**Affiliations:** 1 Faculty of Education and Sport Sciences (Melilla Campus), Department of Didactics and School Organization, University of Granada, Calle Santander, 1, 52071 Melilla, Spain; 2 Faculty of Education Sciences, Department of Didactics and School Organization, University of Granada, Campus Universitario de Cartuja, 18071 Granada, Spain; 3 Faculty of Social and Legal Sciences (Melilla Campus), Department of Applied Economics, University of Granada, Campus de Melilla, Calle Santander, 1, Granada, Spain

**Keywords:** Educational technology, Educational innovation, Training, Learning processes, Teacher education, Teaching skills

## Abstract

The Spanish autonomous city of Melilla, located in northwest Africa, has one of the highest academic failure and abandonment rates in Europe. An effective way to improve this situation would be to improve students’ digital competence. In order to do so, teachers must have competent digital skills themselves and also be able to teach them. To determine teachers’ level of digital competence, the Spanish adaptation of the European Framework for Digital Competence of Educators was used to analyse the self-assessment responses of teachers in training at the Faculty of Education and Sport Sciences in Melilla, Spain. Several quantitative techniques were used to analyse data collected from a questionnaire based on the items in the framework. Indicators were given to each competence using a factor analysis to contrast differences between undergraduate and postgraduate students. Correlations between some of the students’ characteristics and the competences were estimated using OLS. The results show students’ self-assessment level of digital competence in different areas and differences between the bachelor’s and master’s programmes. Digital competence gaps were also detected in teacher training, especially in security. The conclusions highlight the need to improve digital security and facilitate a higher level of digital skills in line with the framework. Indeed, more hours of training in digital competence are required while taking into account the educational context and the technological, pedagogical and content knowledge needed to teach. Equally, the same skills must be developed by educators in order for them to transmit digital competence to their students and support them in educational centres.

## Introduction

This study analyses the gaps in training and training needs of teacher trainees in the city of Melilla in relation to teaching digital competence. The analysis is based on the Common Framework for the Digital Competence of Teachers (CDCFT) (Gabarda et al., 2017; INTEF, 2017), the Spanish adaptation of the European Union’s (EU) European Digital Competence Framework for Educators (DigCompEdu). Improving digital skills training for future teachers would ensure that their students would also acquire digital skills. This, in turn, would improve the educational situation in the city of Melilla (Bejaković & Mrnjavac, 2020; European Commission, 2016; Koliouska & Andreopoulou, 2020; Quaglio et al., 2016; Valarezo et al., 2018) as well as its social (Fang et al., 2019; Garcia-Valcarcel et al., 2014; Reimers, 2020; Wu et al., 2015) and economic (Cruz-Jesus et al., 2016; Reimers, 2020) status. The study of the situation in Melilla could be extrapolated to educational contexts of similar or lesser educational complexity.

Similar studies to assess the digital competence of teachers have been performed in various contexts (Escudero et al., 2019; Ortega-Sánchez et al., 2020; Rojo-Ramos et al., 2020). This study was performed in a complex educational context which has one of the lowest scores in Europe according to the PISA 2015 and other educational reports (Spanish Ministry of Education, Culture and Sport [MECD], 2017). Melilla, located in northwest Africa, has a different social and educational context to other parts of Spain.

Technology has become a major part of our daily personal and professional lives, and has notably improved communications and job performance, among many other aspects (Mendoza et al., 2015; Monllau & Ávila, 2015). However, the use of information and communications technologies (ICT) has also given rise to a new type of social inequality between those who have access to and knowledge of technology and those who do not (i.e., the digital divide), which further deepens existing social inequalities (Blank, 2017; Philip et al., 2017; Ragnedda, 2017; Šuminas et al., 2018; van Deursen & van Dijk, 2019). This new type of inequality is primarily based on economic and educational factors.

Social inequalities related to economic factors can be mitigated by using low-cost devices and opting for the growing trend known as ‘Bring your own device’ (BYOD), which makes the inclusion of technology in the classroom more affordable and sustainable by using devices already available to students (Attewell, 2015; Gkamas et al., 2017; Maher & Twining, 2017). Almost every student has a smartphone, which they can use to reproduce audiovisual material, work on assignments and access emerging technologies such as augmented and virtual reality without incurring extra costs or using additional devices. For example, without using Google Cardboard, you can still play virtual reality content on your smartphone by selecting the full screen option instead of a split screen with separate visuals for each eye and holding the phone in front of your eyes. A lot of free content can be found on the internet such as YouTube VR. Under this scheme, schools should only need to provide a limited number of devices to disadvantaged students to use for educational purposes. Moreover, educational measures and policies are currently promoting the use of ICT in the classroom (Chen et al., 2019; Mahi et al., 2019; Plaza-De La Hoz, 2018; Tairab et al., 2016). Allowing students to use their smartphones via the BYOD initiative increases their motivation to work on assignments and makes it easier for them to access applications and information on the internet (Laxman & Holt, 2017; Maher & Twining, 2017). Consequently, programmes have been devised to help make new technologies available to everyone, regardless of their academic or economic status, thus potentially contributing to bridging the digital divide and inequalities (Pérez-Castro et al., 2021).

Other factors concerning education have also created a new type of inequality i.e., digital illiteracy between those who possess the knowledge and skills needed to use new technologies and those who do not (Cortina-Pérez et al., 2014). Digital illiteracy limits professional development and access to the labour market. To address this phenomenon, the EU has underlined the vital role of education in promoting new technologies. The aim is to strengthen human capital, employability and competitiveness, given that the lack of digital competence increases the risk of unemployment, poverty and social exclusion (Bejaković & Mrnjavac, 2020; Koliouska & Andreopoulou, 2020; Quaglio et al., 2016; Valarezo et al., 2018). To this end, the EU has created a framework of the digital competences (DigCompEdu) citizens need to acquire in order to increase digital literacy across the board (Carretero et al., 2017; Kerkhoff & Cloud, 2020; Pérez-Escoda & Fernández-Villavicencio, 2016). The objective of digital literacy is to facilitate inclusion in the information society, enjoy a fuller and more participatory life, and use essential tools to enhance active citizen engagement (Abad, 2014). The DigCompEdu framework is based on the analysis of other frameworks by Ferrari (2012) to develop twenty-first century skills.

Education policies around the world are currently seeking ways to reduce the digital divide among students. Indeed, ICT is considered a paramount and wide-ranging issue and is included in the objectives of the Horizon 2020 programme (Centre for the Development of Industrial Technology (CDTI & European Office, 2014). The United Nations Literacy Decade (UNLD) and the United Nations Decade of Education for Sustainable Development (UNDESD) both aim to reduce poverty and improve health and quality of life. The two initiatives view education as making an important contribution to the achievement of these goals and are founded on the belief that technologies can help in the process. Moreover, many countries are redefining their education systems to provide twenty-first century skills to support social and economic development (Reimers, 2020).

This study seeks to identify the digital competence needs of Melilla’s teacher trainees in order to improve the city’s educational situation. The city of Melilla has one of the most complex educational contexts in Europe (PISA, 2015), which is a determining factor in the implementation of ICT projects (Mooketsi & Chigona, 2016). Despite the benefits that ICTs bring to society (Fang et al., 2019), people from disadvantaged contexts are excluded from accessing or using them (Tamatea & Pramitasari, 2018). In addition to inadequacies in education, the lack of digital competence also prevents people from being part of society (Wu et al., 2015). As a result, the EU’s Europe 2020 Strategy focuses on the fight against digital inequalities as an attempt to restart Europe’s economy (Cruz-Jesus et al., 2016). The need to improve the level of digital competence in Melilla goes beyond its educational situation, which, due to the complexity of its context, also needs to be studied.

This study is based on the standards set by international organisations such as the European Commission and UNESCO (2008), as well as the Spanish Ministry of Education. The structure and items in the questionnaire have been defined in accordance with Spanish education legislation namely, Organic Law 3/2020, 29 December, which amends Organic Law 2/2006, 3 May on Education, and INTEF guidelines (INTEF, 2017). The items used are the same as those used by the authorities to assess the digital competence of teachers. The difference is in the evaluation, which is assessed by using students’ direct responses on a Likert-4 point scale, as an alternative to presenting evidence for each of the items. This makes the responses dependent on each individual’s self-assessment. The study analyses teacher trainees’ level of self-assessment (Fernández, 2010; Nuere & Díaz-Obregón, 2018) of their own digital competence based on the CDCFT, the Spanish adaptation of the DigCompEdu (Escudero et al., 2019; Ortega-Sánchez et al., 2020; Rojo-Ramos et al., 2020). The participants in this study were all teachers in training pursuing either a BA in Early Childhood Education, a BA in Primary Education, or an MA in Secondary Education at the Faculty of Education and Sports Sciences at the University of Granada Campus in Melilla. Given that this is the only teacher training faculty in Melilla, the study focuses exclusively on the digital competences of a group of teacher trainees in the university of that city. The aim of the study was to determine the strengths and weaknesses of teacher training and identify the competence areas in the CDCFT framework that need to be strengthened (Gimeno & Gallego, 2007). The overall aim is to promote the integration of the latest technological advances into Melilla’s classrooms in order to improve the quality of education and bridge the digital divide (Cruz-Jesus et al., 2016).

## Training teachers in digital competence

Digital competence is based on digital literacy (Martin & Grudziecki, 2006) and digital literacy is attained through acquiring a set of skills and knowledge (Spante et al., 2018). According to the EU communication *Supporting growth and jobs, An agenda for the modernisation of Europe’s higher education systems*, more than two thirds of students and graduates consider that there is a mismatch between their training and the skills demanded by the labour market (Fernández-Cruz & Fernández-Díaz, 2016; Guzmán-Simón et al., 2017). Almost half of higher education teachers share the same opinion and believe there is a need to promote innovation (Guzmán-Simón et al., 2017). Nevertheless, even though teachers recognise the importance of ICT, most state that they only have average personal user skills and below-average digital teaching skills (Falcó, 2017).

There is a gap between digital competence developed in informal learning contexts and that acquired in formal learning contexts such as universities. In general, Spanish universities do not integrate the use of ICT and digital literacy in their training modules (Guzmán-Simón et al., 2017). Some degree programmes, such as education, offer specific courses on ICT, but this is not the case for all degree programmes, despite the fact that new technologies are used in all fields. In turn, support from academic administrations is perceived as limited and most progress is made on an individual basis (Falcó, 2017). This issue is of particular importance given the potential influence that workplace support has on the integration of digital competence (Instefjord & Munthe, 2017). Poor ICT use can also hinder the professional development of teacher trainees, which, once they graduate, can become a source of difficulties in their profession as teachers (Guzman-Simon et al., 2017). Teacher training is therefore of vital importance for successfully introducing new technologies in the classroom (Ramírez-Montoya et al., 2017).

There is also a mismatch between the skills teachers require to develop the digital competence of their students and their own actual skills (Fernández-Cruz & Fernández-Díaz, 2016). Teachers are unable to develop their students’ digital competence if they do not have an advanced command of those skills themselves (Ramírez-Montoya et al., 2017). Neither will they be able to efficiently teach a subject by overcoming isolated pockets of knowledge in technology, content or education without mastering ICT skills (Cabero & Barroso, 2016). This is evidenced by the TPACK framework (Koehler et al., 2014; Mishra & Koehler, 2006) which collects and describes the kinds of knowledge that teachers need relating to Technology (TK), Pedagogy (PK) and Content (CK), including Pedagogical Content Knowledge (PCK), Technological Content Knowledge (TCK), Technological Pedagogical Knowledge (TPK), and Technological Pedagogical Content Knowledge (TPACK). The TPACK framework has proven its effectiveness (Atun & Usta, 2019) by giving less attention to the tool and more attention to the kinds of knowledge, skills, and attitudes needed (KSA) (Mishra & Warr, 2021). The TPACK framework has been explored in numerous studies in the field of education; a number that is currently on the rise (Soler-Costa et al., 2021).

Digital competence gaps in teacher training originate from the very same gaps experienced by the educators who train teachers (Instefjord & Munthe, 2017). If teacher trainees do not recognise their educators as role models in the use of new technologies in education, it is highly unlikely that they will be inspired by them to implement technology in the classroom (Falcó, 2017). Changes in education must therefore involve changes in the methodology used, which requires, among other things, a strategic teacher training plan (Gómez, 2015; Author, 2015). This is where online training of teacher trainees can be used (Perry & Jan, 2017) to focus on the confident, critical and creative use of ICT (Author, 2017; Liang & Fung, 2020). To design such a plan, the teacher trainees’ level of digital competence must first be determined (Guzmán-Simón et al., 2017).

### The benefits of teaching digital competence

Throughout the article the terms ‘competences’ and ‘skills’ are used to refer to different concepts. Skills are often necessary requirements to achieve competences. In this section, we discuss some of the reasons why teachers’ digital competence needs to be developed and outline some of the associated benefits. First, it has already been demonstrated that the application of ICT in education provides many educational benefits (McGarr & Gavaldon, 2018). ICT is currently being used in child and adult education, thus resulting in the on-going development of training tools and programmes (Author, 2020). ICT facilitates the learning process for students, it motivates and helps them become more autonomous. Furthermore, ICT can be adapted to their level, which is especially beneficial for special needs students (García-Valcarcel et al., 2014).

In turn, the use of ICT allows the integration of active methodologies (Gámiz-Sánchez, 2017). Currently, due to the COVID-19 global pandemic, the use of active methodologies has increased, for example virtual teaching (Martín et al., 2021). Active methodologies focus on the student within the teaching–learning process (Jiménez et al., 2020). Innovative methodologies have a long history of excellent results in many educational settings (Fatikhova & Sayfutdiyarova, 2017). Some of the most notable methodologies include flipped classroom learning (Sáez-López & Cózar-Gutiérrez, 2017) and project-based learning (Fatikhova & Sayfutdiyarova, 2017), which is regarded as the ‘superstar’ of methodologies due to its success (Ramírez & Gómez, 2016).

Moreover, ICT is a very powerful motivator that helps students develop responsibility towards others and learning, and also affords the possibly of integrating special needs students (Garcia-Valcarcel et al., 2014). In the acquisition of digital competence, motivation is a key factor and the driving force behind students’ online achievements (Castillo-Merino & Serradell-López, 2014).

The use of ICT also facilitates collaborative learning and the development of twenty-first century skills which have a broader scope than digital competence (van Laar, 2017). This enables the development of social and problem-solving skills, as well as greater autonomy, responsibility and capacity for reflection and initiative (Garcia-Valcarcel et al., 2014), inter alia.

Furthermore, the latest technological and methodological innovations represent an advance in education (Bejaković & Mrnjavac, 2020; European Commission, 2016; Koliouska & Andreopoulou, 2020; Quaglio et al., 2016; Valarezo et al., 2018) that should be used for making improvements in the most disadvantaged socioeconomic contexts. Improvements in citizen education and digital skills would lead to social improvements (Fang et al., 2019; Garcia-Valcarcel et al., 2014; Reimers, 2020; Wu et al., 2015) by facilitating integration in society and the labour and economic market (Cruz-Jesus et al., 2016; Reimers, 2020). Digital competence facilitates access to jobs with higher salaries and new professions, some online or even self-employment. The autonomous city of Melilla, Spain, which is located in the northwest of Africa, is one of the cities with the highest academic failure and abandonment rates in Europe (MECD, 2017) according to recent educational reports, including PISA 2015. In order to successfully implement the latest developments in education, teachers’ digital competence must improve.

## Common digital competence framework for teachers (CDCFT)

The need to establish a body of knowledge that defines digital competence in education has led various international organisations to develop reference guidelines. For example, the study of Computer and Information Literacy (CIL), which was established as a dimension of ICILS 2013 and maintained in ICILS 2018 (Fraillon et al., 2019). In 2008, UNESCO developed the ICT Competency Standards for Teachers (UNESCO, 2008) and has recently disseminated a framework of ICT skills and standards for teacher training in partnership with the Pontificia Universidad Javeriana de Cali (Valencia-Molina et al., 2016). As mentioned, the Common Framework for the Digital Competence of Teachers (CDCFT) is the Spanish adaptation of the EU’s European Framework for the Digital Competence of Educators (DigCompEdu) (Redecker, 2017), which was created to measure and verify the level of educators’ digital competence. In turn, this framework is based on the Common European Framework of Reference for Languages, which classifies language mastery from A1 to C2 levels. Spain has implemented the CDCFT through the National Institute of Educational Technologies and Teacher Training (INTEF, 2017) and divided it into five areas:

### Area 1. Information and data literacy

Locate and retrieve relevant information on the internet and know how to store, organise and analyse such information for its possible applications in teaching. Examples include the creation of teaching materials, presentations, and others.

### Area 2. Communication and collaboration

Share community-created resources and experiences through online tools, allowing feedback between teachers. Belong to teaching communities in social networks. This will enable the dissemination of good practices and the creation of a validated resources bank.

### Area 3. Digital content creation

Create and edit own teaching materials and audio-visual productions, while understanding how copyright and licences are to be applied. In order to provide personalised learning to students, teachers must be able to create their own resources.

### Area 4. Safety

Protect personal data, digital content and use technology in a responsible and safe manner. Problems such as identity theft or cyberbullying may occur if new technologies are not used properly.

### Area 5. Problem solving

Identify needs and know how to choose digital resources for resolving problems. Decide which are the most appropriate tools and use them properly, particularly with regard to active methodologies.

The implementation of the DigCompEdu in Europe has meant that the level of educators’ digital competence can be measured for the very first time (INTEF, 2017). Using the Spanish adaptation, recent studies have been performed to determine the level of digital competence of teacher trainees in Spain (Escoda & Conde, 2016). The results from the self-assessment tests performed by the participants in this study indicate that digital competence is currently lacking in the field of education (Guzmán-Simón et al., 2017). Moreover, teachers’ expectations and beliefs about their own ability have a significant influence on the integration of new technologies (Korthagen, 2017).

The publication of the CDCFT has given rise to various studies aimed at determining teachers’ level of digital competence in order to address ICT shortcomings (Gabarda et al., 2017). However, practical applications have not yet been implemented in schools (Deumal & Guitert, 2015). Several studies have indicated the need for greater emphasis on ICT training to support teaching practices in educational centres (Ramirez-Montoya et al., 2017). The same can be seen in many international publications such as the *Digital Agenda for Europe* (European Commission, 2013), the *TALIS* (OECD, 2014) and the *ICSL* reports (Valle et al., 2015).

In order for teachers to implement the framework in the classroom, other factors, in addition to technology, must also be taken into account. This can be seen in the TPACK model (Koehler et al., 2014; Mishra & Koehler, 2006) and the Will, Skill, Tool (WST) model of technology integration (Knezek et al., 2000), which postulates that will (positive attitudes), skill (technology competency), and tool (access to technology tools) are all necessary components for teachers to effectively integrate information technology into classroom practices (Agyei & Voogt, 2011).

## Methodology

A set of quantitative methods were used to analyse the data collected from a questionnaire based on the items in the CDCFT published by the INTEF. For this study, all the items from the five competence areas in the CDCFT, shown in the appendices, have been taken into account. Our analysis consists of several stages. First, we created an indicator for each competence area using item factor loadings derived from an exploratory factor analysis (Hair et al., 2009; Härdle & Simar, 2012). Second, we performed a descriptive analysis of the indicators using the main descriptive statistics and created several boxplots. Next, we used statistical inference to test whether there were differences between the groups proposed in the second stage. To this end, we previously checked the assumption of normality using the Kolmogorov–Smirnov test with the Lilliefors correction (1967). In this case, if the assumption is fulfilled, we use the *t*-test, and if not, we apply the nonparametric alternative Mann–Whitney–Wilcoxon test. And lastly, we estimated a multiple linear regression model using ordinary least squares (OLS) to determine the relationship between the indicators and the independent variables of interest such as the degree being studied, the academic year or the teacher trainee’s gender (Gujarati et al., 2012; Wooldridge, 2016). This array of techniques enables researchers to describe the current situation (Best, 1970) and makes it easier to explain the subject matter (Verma & Mallick, 1999). This is a non-experimental study, in which the results were observed and then analysed in order to respond to the issues raised.

### Objective

The aim of the study was to determine the level of digital competence of teacher trainees studying the BA in Early Childhood Education, the BA in Primary Education, and the MA in Secondary Education at the Faculty of Education and Sports Sciences at the University of Granada Campus in Melilla. To achieve this objective, the self-assessed level of digital competence in response to the items in the CDCFT was analysed.

#### Hypothesis

Improving digital skills training for future teachers would ensure that their students would also acquire digital skills. This, in turn, would improve the educational situation in the city of Melilla and, subsequently, its social and economic status.

#### Objective

To analyse the training needs and gaps in digital competence training for teachers in training in the city of Melilla.

### Sample

A total of 266 students (early childhood education 68, primary education 154 and master’s 44) were invited to participate in the study and 176 accepted (early childhood education 46, primary education 110 and master’s 20). The average age of the invited sample is 21. The total sample is 176; 154 face-to-face responses and 22 online responses (5 BA in Early Childhood Education, 10 BA in Primary Education and 7 MA in Teacher Training for Compulsory Secondary Education, Baccalaureate, Professional Training and Teaching of languages). The entire student body consisted of the three degree-programmes mentioned above (two bachelor’s degrees and one master’s degree in education). In order to ensure the sample was representative of the population, it covered four academic years and included students from the three degree-programmes. The accepting sample is shown in Table 1 for each of the academic years and degrees.

**Table 1:** Descriptive statistics: explanatory variables

Variable	Mean	SD	Min	Max
Gender	0.7614	0.428	–	–
Degree_1	0.625	0.486	–	–
Degree_2	0.1136	0.3182	–	–
Year	2.097	1.062	1	4
Age	22.28	3.903	18	40

Degree_1 refers to the bachelor’s programme in primary education, while Degree_2 refers to the master’s programme

Table 1 shows the main descriptive statistics of the explanatory variables. It can be observed that of the 176 teacher trainees that comprise the total sample, 62.5% were enrolled in a BA in Primary Education, 11.36% in an MA in Secondary Education and 26.14% in a BA in Early Childhood Education. Additionally, 76% of the sample were female (the variable ‘gender’ takes the value of 1 if female and 0 otherwise) and the average age was 22.28 years old. Finally, it should be noted that 39.21% of those surveyed were in their first academic year, 24.43% in their second year, 23.86 in their third year and 12.5% in their fourth year.

### Measurement

For this study, we used a questionnaire that measures the teacher trainees’ level of digital competence based on the CDCFT. The questionnaire is divided into the five areas within the aforementioned framework and contains 91 items distributed over the five areas (Table 2):

- Competence area 1: Information and data literacy (16 items)
- Competence area 2: Communication and collaboration (31 items)
- Competence area 3: Digital content creation (16 items)
- Competence area 4: Safety (13 items)
- Competence area 5: Problem solving (15 items)

**Table 2:** Items on the questionnaire used for the data collection by competence area

Questionnaire Items By Area
*Area 1*
1.1. Browsing, searching and filtering data, information and digital content
1.2. Evaluating data, information and digital content
1.3. Managing data, information and digital content
*Area 2*
2.1. Interacting through digital technologies
2.2. Sharing through digital technologies
2.3. Engaging in citizenship through digital technologies
2.4. Collaborating through digital technologies
2.5. Netiquette
2.6. Managing digital identity
*Area 3*
3.1. Developing digital content
3.2. Integrating and re-elaborating digital content
3.3. Copyright and licences
3.4. Programming
*Area 4*
4.1. Protecting devices
4.2. Protecting personal data and privacy
4.3. Protecting health and well-being
4.4. Protecting the environment
*Area 5*
5.1. Solving technical problems
5.2. Identifying needs and technological responses
5.3. Creatively using digital technologies
5.4. Identifying digital competence gaps

A type of Likert 4-point scale was used to measure the responses. Respondents assigned a value to various statements on the use of a variety of technological aspects according to their knowledge: 1 ‘Strongly disagree’, 2 ‘Disagree’, 3 ‘Agree’ and 4 ‘Strongly agree’.

All statements refer to the mastery of different aspects of technology. Consequently, the more respondents agree with a statement, the greater their mastery of that aspect of technology, while the less they agree, the lower their overall competence.

### Procedure

Permission was sought from the teachers of the early childhood education and primary education degrees, the master’s degree and the faculty to perform the survey. The questionnaire was administered in the classrooms of the teacher trainees in their respective subjects. First, a brief explanation was given to the respondents regarding the objectives of the research, as well as on the CDCFT. Any doubts were then resolved before giving the questionnaire to the teacher trainees who had previously agreed to participate in the survey voluntarily and signed an informed consent form.

In order to ensure participation in the study by the greatest possible number of teacher trainees, an online version of the questionnaire was also made available by e-mail. This allowed the trainees who were absent on the day of the survey to participate.

Once the data was collected, it was processed using the RStudio 1.4.1106, R 4.0.3, and the car packages, lmtest and nortest.

## Results

In what follows, we discuss the results of the respondents’ self-assessment of their level of digital competence as measured in the questionnaire on the five CDCFT competences.

First, an indicator for the teacher trainees’ level of self-assessment was defined for each competence area based on the assessment of the items. The indicators were constructed as a weighted average using the factor loading of the item in its competence area to determine the weight. The factor loadings were calculated using the principal component extraction method (Hair et al., 2009; Härdle & Simar, 2012). The indicators were constructed this way in order to assign a value to each item according to its contribution to the competence. The factor loadings for each indicator are shown in detail in the appendices.

Second, we performed a descriptive analysis of the indicators. The main descriptive statistics for the indicators are shown in Table 3. No significant variations were observed in the mean and the standard deviation between the indicators. Specifically, the mean for the indicators ranged from 2.75 to 3.10, while the standard deviation ranged from 0.48 to 0.55.

**Table 3:** Descriptive statistics of the indicators

Indicator	Mean	SD	Min	Max	Skewness	Kurtosis	Cronbach’s alpha
Competence area 1: Information and data literacy	3.098	0.482	1.610	4	−0.416	−0.291	0.870
Competence area 2: Communication and collaboration	2.745	0.484	1.579	4	0.026	−0.356	0.900
Competence area 3: Digital content creation	2.873	0.511	1.758	4	0.006	−0.811	0.854
Competence area 4: Safety	2.943	0.551	1.508	4	−0.254	−0.400	0.723
Competence area 5: Problem solving	2.749	0.527	1.469	4	0.201	−0.461	0.830

The coefficients of skewness and kurtosis are close to zero in all cases, which indicates that the distribution of the competence area are almost symmetric and mesokurtic. In relation to the reliability of the indicators, we obtained high Cronbach’s alpha scores. All the indicators have values higher than 0.8, which is considered good, with the exception of Competence Area 4, which is higher than 0.7 and is considered acceptable (Cronbach, 1951).

**Fig. 1:**
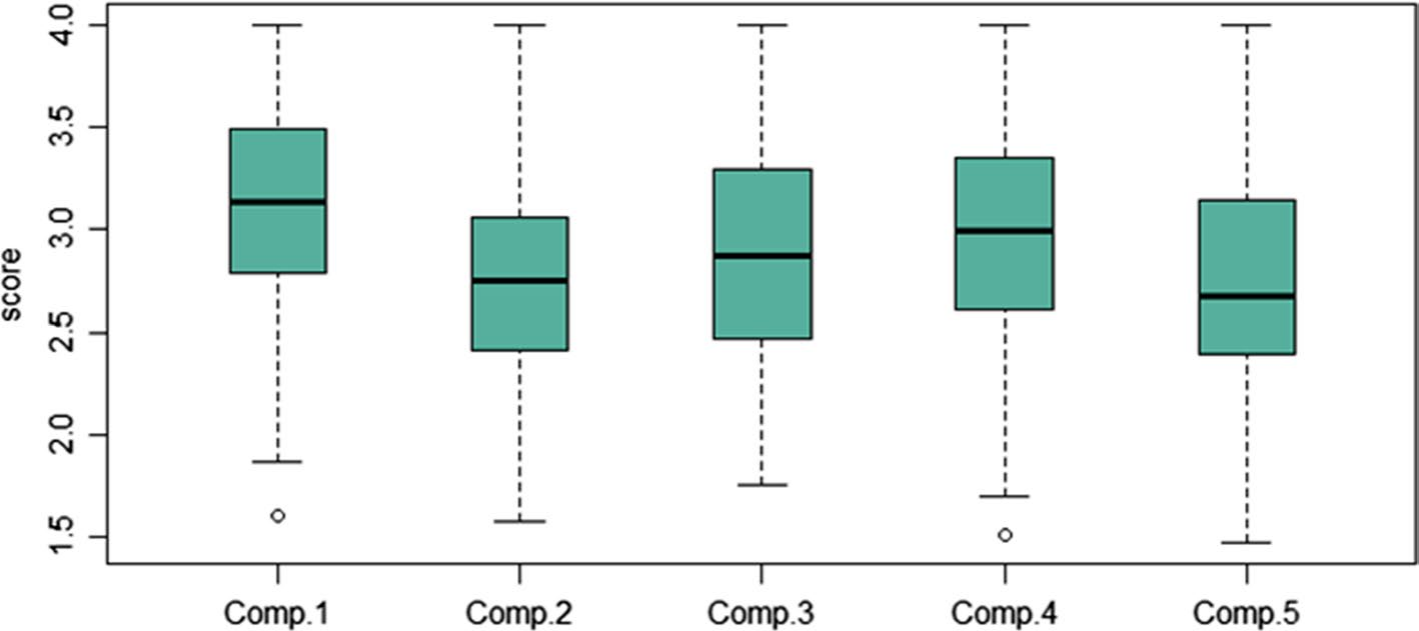
Boxplot of indicators by competence area

**Fig. 2:**
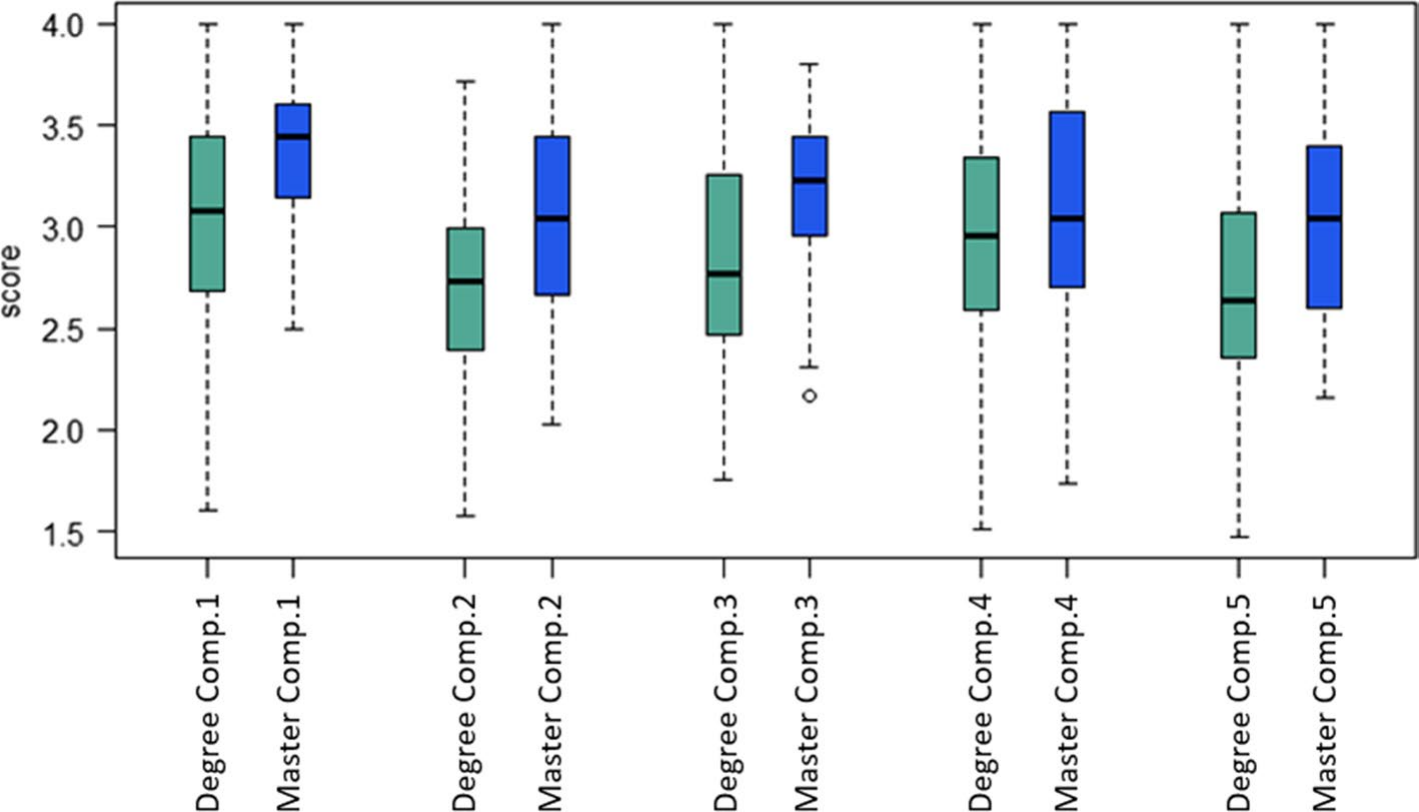
Boxplot of indicators by competence area grouped according to undergraduate and postgraduate degrees

Figures 1 and 2 show the distribution of the indicators. As can be observed in Fig. 1, there are no major variations between competence areas. In other words, all the indicators have an almost symmetrical distribution with values ranging from 1.6 to 4. Worthy of note is the indicator for Competence Area 1, which shows a greater degree of skewness. Specifically, 25% of the trainees who obtained the highest values for the indicator are in the range 3.5–4, while the 25% who obtained the lowest values are in the range 1.6–2.8. The indicator for Competence Area 4 shows a similar distribution, although it is less accentuated. The 25% of trainees with the highest values for the indicator are in the range of 3.35–4, while the 25% with the lowest values are in the 1.5–2.6 range. Moreover, the middle 50% of values for the indicator of Competence Area 5 are not distributed homogeneously and there is a greater distance between the median (2.7) and third quartile (3.2) compared to the first quartile (2.4). Two outliers with particularly low values can also be observed for Competence Areas 1 and 4.

Figure 2 shows the box and whisker plot for the indicators grouped by type of degree (i.e., bachelor’s or master’s). As can be observed, students studying for their master´s degree have higher skill acquisition values and a higher median value in all the indicators, while the undergraduates show a lower minimum value. For example, in the distribution of the indicator for Competence Area 3 (Digital content creation), the values for the master´s degree students in comparison to the undergraduates’ values are more concentrated, although the maximum value is higher for the undergraduate students.

The relationship between degree programme and academic year for the indicators of the competence areas is analysed below. Specifically, OLS was used to fit a regression line between each indicator, the explained variable, and the explanatory variables degree and academic year. The variables age and gender were also included. This fit method was chosen because of the advantages it presents with respect to other methods, namely the easy and intuitive interpretation of the regression coefficients.

As mentioned, the sample comprised students from two undergraduate degree programmes and one postgraduate degree programme: the BA in Early Childhood Education, the BA in Primary Education and the MA in Secondary Education. In order to include this information in the model, two dummy variables were used: Degree_1 and Degree_2. These variables take the value 1 if the respondent was a primary education or a master’s degree student, respectively, and 0 otherwise. When both variables take the value 0, it means that the respondent is an early childhood education student.

In the third stage of the analysis, we contrasted the differences in means for the groups set in the previous stage. Table 4 shows the results of the analysis of discrepancies on the averages. Furthermore, normality tests were included to verify the initial assumption before applying the *t*-test. If the assumption is not satisfactory, then the nonparametric alternative Mann–Whitney–Wilcoxon is shown. The normality test is significant only in Competence Area 5 for the BA degree group. In general, it was observed that the differences are significant between groups. Competence Area 4 is the exception.

**Table 4:** Analysis of discrepancies of means in undergraduate and master students

	K-S group Degree	K-S group Master	*t*-test
Comp. 1	0.062 (0.1756)	0.110 (0.7544)	−3.9402 (0.0004)
Comp. 2	0.045 (0.6234)	0.125 (0.5693)	−2.8181 (0.0097)
Comp. 3	0.086 (0.2117)	0.150 (0.2825)	−2.5923 (0.0157)
Comp. 4	0.043 (0.7138)	0.142 (0.3604)	−0.9772 (0.3384)
Comp. 5	0.075 (0.0333)	0.104 (0.8297)	1027.5* (0.0172)

Contrast statistic and *p *value between parenthesis *test Mann–Whitney–Wilcoxon

Table 5 shows the results of the regressions performed on each indicator. It should be noted that the objective of the regressions was to establish relationships between some of the trainees’ characteristics and the perceived acquisition of skills. In general, the variables ‘degree’ and ‘academic year’ were found to be significant in the models, while the variables ‘age’ and ‘gender’ were not significant for any of the indicators.

**Table 5:** Results of the regressions

	Comp. 1	Comp. 2	Comp. 3	Comp. 4	Comp. 5
(Intercept)	2.997^*^	2.462^*^	2.747^*^	2.700^*^	2.663^*^
	(0.000)	(0.000)	(0.000)	(0.000)	(0.000)
Degree_1	0.094	0.167^*^	0.015	0.195	0.145
	(0.265)	(0.049)	(0.871)	(0.056)	(0.122)
Degree_2	0.603^*^	0.610^*^	0.453^*^	0.348^*^	0.614^*^
	(0.000)	(0.000)	(0.005)	(0.0472)	(0.000)
Age	−0.014	−0.008	−0.006	−0.003	−0.014
	(0. 214)	(0.509)	(0.645)	(0.811)	(0.271)
Gender	0.079	0.046	−0.032	0.034	−0.012
	(0.369)	(0.595)	(0.731)	(0.748)	(0.905)
Year	0.108^*^	0.114^*^	0.102^*^	0.061	0.115^**^
	(0.008)	(0.005)	(0.019)	(0.205)	(0.010)
R^2^	0.111	0.122	0.072	0.041	0.095
Num. obs	168	172	175	168	172
RMSE	0.4601	0.4602	0.4995	0.5491	0.5106

Degree_1 refers to the bachelor’s programme in primary education, while Degree_2 refers to the master’s programme. ^*^p < 0.05. The *p* values are shown in parentheses

Specifically, a positive relationship was found between master’s degree students and the value of the indicator for the competence area. For example, in Competence Area 1 (Information and data literacy), master’s degree students obtained a mean value of 0.603, which is higher than the value for the bachelor’s degree students in early childhood education. Similar results were also observed for the rest of the indicators. Significant differences were also found in Competence Area 2 (Communication and collaboration) among the two groups of students. In particular, the primary education students obtained a mean value of 0.167, which was higher than the early childhood education students.

In almost all cases, the ‘academic year’ variable is positively correlated with the indicator of the corresponding competence area. The effect of being in a later academic year has a positive influence on the indicator of the competence area. Competence Area 5 (Problem solving) presents the greatest variation with an increase of 0.115 per academic year.

## Discussion and conclusions

In general, teacher trainees perceive a progressive improvement in their digital competence throughout their study programmes. In other words, the higher the level of education they receive, the more positive their self-assessment (Gómez, 2015; Author, 2015). The results for the master’s degree students are higher than those of the fourth-year students taking the early childhood education and primary education degrees. This indicates that from the time the students complete the bachelor’s degree to the time they complete the master’s degree, there is an improvement in the ICT skills acquired. This improvement in ICT skills may also be due to the students’ own experience or self-training during this same time period (Instefjord & Munthe, 2017). Therefore, the higher the academic level of the respondent, the higher the level of self-assessed digital competence.

In contrast, the early childhood education students obtained the lowest values. More specifically, statistically significant differences were found between the early childhood education students and the primary education students in their digital competence values. This could be due to the fact that the study programmes provide teacher trainees at higher educational levels with more training in digital competencies than those at lower levels, and that lower levels require a lower level of digital competence to perform their job functions (Ramírez-Montoya et al., 2017). These differences might also be explained by the fact that teacher trainees who will be teaching at higher educational levels are more concerned about acquiring better digital competence (Falcó, 2017).

In general, the learning outcomes were positive, given that the values for the acquisition of competences increase as students advance in their degree. The exception to this is Competence Area 4 relating to safety (INTEF, 2017); this value does not increase as students progress over the years (Gómez, 2015). However, the value remains quite high throughout the years, which might suggest that the teacher trainees already possess this knowledge and are not provided with new content to add to what they already know (Guzmán-Simón et al., 2017). Nonetheless, it is essential to make teachers aware of the importance of using new technologies in a critical and responsible way that respects both the health and safety of users and the environment (Redecker, 2017; Simandl et al., 2017).

As mentioned above, teachers must have a minimum level of digital competence in order to use technologies and methodologies in an appropriate manner when teaching specific content and to adapt to the educational context and resources at their disposal (Cabero & Barroso, 2016; Guzmán-Simón et al., 2017). In addition to technological knowledge (TK), teachers also need to have content knowledge (CK) of what is to be taught and pedagogical knowledge (PK) in order to teach it correctly (TPACK model) (Atun & Usta, 2019). They must also have technology-related knowledge, skills and attitudes (KSA) (Mishra & Warr, 2021). This is of particular importance in complex educational settings, such as socioeconomically disadvantaged situations (Blank, 2017; Philip et al., 2017; Ragnedda, 2017; Šuminas et al., 2018; van Deursen & van Dijk, 2019) and those characterised by high academic failure and abandonment rates where teaching is more difficult, as in the case of Melilla (MECD, 2017).

Despite the fact that technological resources should be used in all teacher training courses and although there is a specific compulsory subject on ICT learning in the third year of the early childhood education degree and in the second year of the primary education degree, the master’s degree does not offer a specific course on the use of ICT in education. However, ICT training may be insufficient (Guzmán-Simón et al., 2017) if the educators who provide it do not continue to be trained themselves (Redecker, 2017). It creates a mismatch between training and the skills demanded by the labour market (Fernández-Cruz & Fernández-Díaz, 2016; Guzmán-Simón et al., 2017). Hence, the acquisition of digital competence should be regarded as lifelong learning (Redecker, 2017) so that teachers are always up to date. To make ICT training more flexible and accessible, it could be provided online (Perry & Jan, 2017). This would put the focus on universities to educate teacher trainees and guarantee the skills of the educators who train them, thus providing workplace support (Instefjord & Munthe, 2017). The principal factor in achieving educational change in the classroom is the teacher and in order to educate digitally competent teachers, the focus must be on those who train them (Instefjord & Munthe, 2017).

In conclusion, this study analysed teacher trainees’ level of digital competence based on the items in the CDCFT (Gabarda et al., 2017; INTEF, 2017). According to the analyses, the most relevant results are as follows. As the number of years in higher education increases, teacher trainees’ positive self-assessment of their digital competence also increases in all areas except Competence Area 4, safety (INTEF, 2017). This highlights the progressive training of students in new technologies throughout their education (Gómez, 2015). Master’s degree students have a more positive self-assessment of their digital competence in all areas than students in the early childhood and primary education degrees. Again, this demonstrates that the higher the level of education, the higher the level of digital competence according to the students’ self-assessment (Redecker, 2017). Experience should also be taken into account in the case of master’s degree students. Many of them had completed their degree and were working, having received some additional training.

The results point to the need for more, improved training in digital competence (Escudero et al., 2019; Ortega-Sánchez et al., 2020; Rojo-Ramos et al., 2020). In the case of Melilla, digital competence could focus on socio-economically disadvantaged contexts given that, if used wisely, the cost of technology could be minimised (Attewell, 2015; Gkamas et al., 2017; Maher & Twining, 2017). Digital competence in the classroom is dependent on the training received by teachers (Gómez, 2015). Consequently, educators must take into account not only the context (Mooketsi & Chigona, 2016), but also the technological, pedagogical and content knowledge they need to teach (Koehler et al., 2014; Mishra & Koehler, 2006). They must also encourage teachers to acquire the necessary knowledge, skills and attitudes they must have in order to fulfil their role (Agyei & Voogt, 2011; Knezek et al., 2000; Korthagen, 2017; Mishra & Warr, 2021). In order for teachers to stay up-to-date, training must be ongoing throughout their careers (Redecker, 2017), in person or online (Perry & Jan, 2017), to facilitate training as is currently the case due to the COVID-19 global pandemic (Martín et al., 2021). University professors must also be included in training programmes in order to train future professors (Instefjord & Munthe, 2017).

Training must also be reinforced in the area of digital security (Redecker, 2017; Simandl et al., 2017) given that a certain score must be obtained in all areas in order to attain the acceptable level of digital competence set by the CDCFT framework, (INTEF, 2017). For example, if a student obtains level C2 in all areas except security in which they have an A2, then their digital competence level would be A2 because that is the level they have mastered in all areas. Currently, the level of digital competence of the teacher trainees in this study is hampered by digital security. By improving their competence in this area, they would be able to obtain a higher level of digital competence (INTEF, 2017).

By implementing these measures in educational centres (Instefjord & Munthe, 2017), the digital competence of teachers in training in Melilla would improve and gaps in their skills would be eliminated when teaching their students (Falcó, 2017; Fernández-Cruz & Fernández-Díaz, 2016). Moreover, the shortcomings in university digital competence education in teacher training programmes would also improve (Guzmán-Simón et al., 2017). If future teachers adhere to these measures, then they will be able to correctly train their students in digital competence when teaching classes in schools, thus improving the digital competence of the population of Melilla. This, in turn, would also improve the development of the city (Bejaković & Mrnjavac, 2020; Koliouska & Andreopoulou, 2020; Quaglio et al., 2016; Valarezo et al., 2018; van Laar, 2017), not only its overall level of education (Cabero & Barroso, 2016; McGarr & Gavaldon, 2018; Ramírez-Montoya et al., 2017), but also its economic (Cruz-Jesus et al., 2016; Reimers, 2020) and social (Fang et al., 2019; Garcia-Valcarcel et al., 2014; Reimers, 2020; Wu et al., 2015) status.

The recent implementation of the Common Framework for Digital Competence for Teachers (INTEF, 2017) also shows that more research is needed in this field, in particular focusing on specific strategies that need to be implemented (Deumal & Guitert, 2015). This process of change should be based on the confident, critical and creative use of ICT for the acquisition of digital competence (Redecker, 2017; Simandl et al., 2017).

## Limitations and future research

This study has several limitations. First, although the data collected in the questionnaire indicate a high level of digital competence among the respondents in the three degree-programmes, they reflect the respondents’ own self-assessment, so their level of perceived digital competence may not coincide with their actual level (Korthagen, 2017). The subjectivity of respondents regarding their knowledge and level of mastery is one of the main issues with self-assessment. However, self-assessment is still a valid tool for ascertaining how students perceive their own learning (Fernández, 2010; Nuere & Díaz-Obregón, 2018), and enables us to detect their strengths and weaknesses (Gimeno & Gallego, 2007). Second, the CDCFT items do not measure the respondents’ knowledge of methodologies, which is a fundamental factor for teaching new technologies (Cabero & Barroso, 2016). Technology on its own is merely a tool and must be combined with the appropriate methodology if it is to be used successfully in the classroom (O'Flaherty & Phillips, 2015). Third, the study does not measure the real capacity of teacher trainees to apply their knowledge in practice; a factor that is especially important in difficult educational contexts, such as the case of Melilla. This would involve, among other things, adapting technology to make it affordable in order to overcome one of the main obstacles of the digital divide (Wu et al., 2015).

Future research should therefore analyse teacher trainees’ educational digital competence to determine the gap between their self-assessed and actual competences. Teachers should be assessed in real educational situations to ensure that they are capable of disseminating their knowledge. It would also enable us to compare the students’ level of self-assessment of their knowledge from this study with their actual knowledge. Research could also be performed on the use of active methodologies and new technologies in a practical and contextualised way to obtain more precise results on the real possibilities of implementing ICT in Melilla and other educational contexts.

## Implications of the Study

The results of the study provide knowledge about the shortcomings detected in a very particular and complex type of educational context and offer alternatives on how to address issues. Furthermore, it enables new research to continue to explore the same topic in greater depth through new experiments or by generating knowledge through comparison with similar studies. In other words, it contributes to the theoretical basis of future research.

The most immediate practical implication is to implement the conclusions of the study to improve the educational and social situation in the city of Melilla. Similarly, the knowledge obtained can be applied to other contexts of similar or lesser complexity. The practical implementation may give rise to new research that analyses the evolution of similar educational and social contexts in greater detail from the perspective of improvements in the educational situation and factors that differ from other contexts.

## Appendix 1

Table 6 shows the factor loadings for each of the items in relation to their corresponding competence. The factor loadings were calculated by principal components extraction and used to construct the indicators for the five competence areas.

**Table 6:** Item factor loadings

Item	Comp. 1	Item	Comp. 2	Item	Comp. 3	Item	Comp. 4	Item	Comp. 5
A111	0.473	A211	0.486	A311	0.547	A411	0.644	A511	0.580
A112	0.599	A212	0.392	A312	0.584	A412	0.674	A512	0.645
A113	0.584	A213	0.400	A313	0.528	A413	0.626	A513	0.698
A114	0.620	A214	0.560	A321	0.638	A421	0.629	A521	0.268
A115	0.659	A215	0.628	A322	0.556	A422	0.632	A522	0.554
A121	0.572	A221	0.647	A323	0.586	A423	0.116	A523	0.515
A122	0.633	A222	0.616	A324	0.582	A431	0.562	A531	0.482
A123	0.557	A223	0.512	A325	0.578	A432	0.672	A532	0.637
A124	0.520	A224	0.394	A331	0.561	A433	0.547	A533	0.627
A125	0.515	A225	0.517	A332	0.634	A434	0.541	A534	0.602
A126	0.633	A226	0.597	A333	0.490	A441	0.669	A541	0.175
A131	0.652	A231	0.567	A334	0.504	A442	0.674	A542	0.581
A132	0.601	A232	0.571	A341	0.559	A443	0.653	A543	0.413
A133	0.583	A233	0.580	A342	0.657			A544	0.673
A134	0.605	A234	0.669	A343	0.406			A545	0.532
A135	0.582	A241	0.594	A344	0.635				
		A242	0.594						
		A243	0.584						
		A244	0.694						
		A245	0.597						
		A251	0.531						
		A252	0.417						
		A253	0.454						
		A254	0.415						
		A255	0.301						
		A261	0.480						
		A262	0.196						
		A263	0.171						
		A264	0.348						
		A265	0.281						
		A266	0.466						

## Appendix 2

### Questionnaire items

1. Competence area 1: Information and data literacy.

1.1. Browsing, searching and filtering data, information and digital content.

**Table:**

1.1.1	I search the internet to find information and resources for educational purposes
1.1.2	I select and adapt the different types of digital resources and information I find on the internet to my training needs
1.1.3	I analyse the information and resources I find on the internet and filter them according to my training needs
1.1.4	I am able to manage the information flows I access (HR, subscriptions, etc.) for my teacher training and share the information with my colleagues
1.1.5	I use keywords and specific vocabulary, sometimes in English, to find the information I need

1.2. Evaluating data, information and digital content.

**Table:**

1.2.1	I assess the suitability and quality of the resources and information I find on the internet for my training
1.2.2	I critically evaluate the resources and information I find on the internet and use in my training
1.2.3	I analyse the reliability of several sources of information before using them
1.2.4	I use strategies to evaluate the usefulness and accuracy of the resources and information I find on the internet in order to optimise the time spent searching for them
1.2.5	I am aware of digital media copyrights and licences and evaluate them before using them in my training
1.2.6	I develop and use advanced search strategies to find information and resources on the internet and share them with my colleagues

1.3. Managing data, information and digital content.

**Table:**

1.3.1	I am familiar with and use the existing storage systems on different devices
1.3.2	I use both local and online storage strategies (applications and browser extensions) to save files related to my training
1.3.3	I update resources and information related to my training, create copies of them and store those I do not use
1.3.4	I have cloud or external storage devices where I store and share resources and files that may be of interest
1.3.5	I am able to compress files and create backup copies of the materials I use to optimise the storage space I have on my devices

2. Competence area 2: Communication and collaboration.

2.1. Interacting through digital technologies.

**Table:**

2.1.1	I am aware of the number of existing online applications and communication tools and use them to help me achieve my training objectives
2.1.2	I communicate with my colleagues and teachers through various online communication services where I exchange information on my teacher training
2.1.3	I am able to create and manage communication networks between my colleagues where we communicate and share files related to our training
2.1.4	I am aware of the existence of educational social networks, and I actively collaborate and participate in them
2.1.5	I design and use different communication and file transfer strategies depending on the audience and the activity

2.2. Sharing through digital technologies.

**Table:**

2.2.1	I analyse and critically evaluate the digital educational information I have access to, share it and encourage my peers to do the same
2.2.2	I interact for educational purposes through my applications and web spaces with students in my degree and with teachers by publishing news and information on education
2.2.3	I regularly redistribute and publish educational information that I consider relevant to our training
2.2.4	I keep a critical and tolerant attitude in social spaces for online communication and I am aware of the cultural diversity that exists in them
2.2.5	I collaborate and cooperate via internet with members of the educational community and use ICTs in a responsible and effective manner
2.2.6	I use online spaces to share educational content I consider relevant for my community of followers and provide feedback and recommendations

2.3. Engaging in citizenship through digital technologies.

**Table:**

2.3.1	I participate in online spaces where I search and read documents to improve teaching practice and I publish opinions about the topic
2.3.2	I have a digital signature and use the digital environments provided by public services
2.3.3	I frequently use my digital devices to do educational administrative procedures online
2.3.4	I participate and do procedures in virtual spaces related to digital citizenship that are useful for my future teaching profession

2.4. Collaborating through digital technologies.

**Table:**

2.4.1	It is easy for me to access and participate in online spaces or share documents online
2.4.2	I feel confident in using online applications and collaborative spaces for my future work as a teacher
2.4.3	I create, share and comment on online documents with educational content that can help my colleagues
2.4.4	I access and participate in online spaces to which I have been invited and where I create and share educational documents with other participants
2.4.5	I participate in online discussions about education, as I believe it is important to foster the development of intercultural awareness and values when working in shared digital spaces

2.5. Netiquette.

**Table:**

2.5.1	I am aware of the existence of norms and conventions for online writing and communication (netiquette) and use them consciously
2.5.2	I write messages and use the various forms of online communication in a respectful and non-offensive manner
2.5.3	I am aware of the main risks to students of using the internet (cyberbullying) and have information on how to detect and act upon such risks in the event they occur
2.5.4	I have information on the risks and inappropriate uses of the internet and am training to address problems of this type that may arise in my educational practice
2.5.5	I believe it is essential to develop intercultural awareness and respect among students and promote the use of netiquette by them

2.6. Managing digital identity.

**Table:**

2.6.1	I know about and understand the concepts of digital identity and digital reputation and I care about and value them in my networks
2.6.2	I care about and protect the image I project in networks by avoiding posting images or videos that could damage my personal reputation
2.6.3	I provide my personal data only on secure network sites and easily identify deceptive messages and/or scams
2.6.4	I create secure keys or passwords that I regularly change as a protection protocol
2.6.5	I am aware of the importance of managing my digital identity as a future teacher
2.6.6	I can easily manage my accounts from any device at any time and know what cookies are and how to manage them

3. Competence area 3: Digital content creation.

3.1. Developing digital content.

**Table:**

3.1.1	I use online tutorials to learn how to use applications for creating digital educational content
3.1.2	I am familiar with and use word processing and presentation software on my digital devices during my training
3.1.3	I use image, video and audio editing software to adapt audiovisual material to my training needs

3.2. Integrating and re-elaborating digital content.

**Table:**

3.2.1	I search for and use materials and programs that could be useful educational resources for me
3.2.2	I edit, adapt and/or collaboratively create digital educational resources for my students
3.2.3	I have a public or private space where I store digital educational resources or materials
3.2.4	I store resources or files in an organised way in my devices that can be useful for my future teaching practice
3.2.5	I am able to plan teaching activities using digital resources and adapt them to my needs

3.3. Copyright and licences.

**Table:**

3.3.1	I know the difference between the various types of licences, and I correctly cite the source of all the digital content I use
3.3.2	I am aware of, respect and use different types of licences in my teaching practice
3.3.3	I search for information about how to properly cite copyrighted content to ensure it is used correctly
3.3.4	I promote and support the notion that educational centres provide free access to knowledge

3.4. Programming.

**Table:**

3.4.1	I know how the internet and some programming software for certain digital applications work
3.4.2	I update my computer and technology knowledge regularly and I am aware of the potential of artificial intelligence in education
3.4.3	I am able to create and program simple educational video games using various tools
3.4.4	I search for solutions to various problems that may arise in computer and educational technology processes in an autonomous way

4. Competence area 4: Safety.

4.1. Protecting devices.

**Table:**

4.1.1	I understand the risks for my digital devices when accessing certain websites and try to avoid such risks using different strategies
4.1.2	I avoid risks associated with using cloud-based tools and accessing certain websites by using and changing my passwords on those sites
4.1.3	I use advanced and updated protection software (antivirus, etc.) in all my devices

4.2. Protecting personal data and privacy.

**Table:**

4.2.1	I am aware of the risks of using the internet and I apply personal data protection strategies (e.g., never repeat the same passwords)
4.2.2	I safely store and retrieve different access data to my accounts
4.2.3	I encourage the responsible use of technology, as well the respect and protection of personal digital data using the appropriate privacy settings according to my objectives

4.3. Protecting health and well-being.

**Table:**

4.3.1	I am aware of the physical and psychological risks derived from the incorrect use of technology and I apply prevention strategies by managing my emotions when certain problems arise
4.3.2	I develop intervention patterns to be executed daily in order to avoid cyberbullying and reinforce safety on the internet
4.3.3	I monitor the time spent working online and apply the correct ergonomic postures to avoid any physical harm
4.3.4	I am aware of the intervention protocols in cases of addiction to technology

4.4. Protecting the environment.

**Table:**

4.4.1	I apply energy saving measures to limit the impact of technologies on the environment
4.4.2	I use technologies in a sustainable manner by promoting the recycling and reuse of disused equipment
4.4.3	I apply recommendations to reduce consumption of supplies (hardware, ink, paper) in order to lessen the carbon footprint

5. Competence area 5: Problem solving.

5.1. Solving technical problems.

**Table:**

5.1.1	I try to individually solve common technical problems related with my digital devices with the support of tutorials
5.1.2	I have sometimes solved technical problems related with my digital devices through online communication
5.1.3	I solve less common technical issues related with digital devices and environments that I use in my training

5.2. Identifying needs and technological responses.

**Table:**

5.2.1	I know some tasks that can be done by using technologies for the improvement of teaching and learning
5.2.2	I take online courses and participate in virtual training spaces of different sorts that promote autonomous learning
5.2.3	I search and use digital tools and applications to solve problems and needs in my training

5.3. Creatively using digital technologies.

**Table:**

5.3.1	I create and use different means of digital expression (blogs, posters, websites, etc.)
5.3.2	I attend online social events to share educational experiences or search for innovative solutions to various problems related to education
5.3.3	I have participated in some innovative digital projects
5.3.4	I plan and participate on virtual environments in the creation of digital educational content

5.4. Identifying digital competence gaps.

**Table:**

5.4.1	I am aware that I have to improve my digital competence and know my limitations in this area
5.4.2	I know the latest improvements related to digital competence and try to keep myself up-to-date in an autonomous way
5.4.3	I mainly use the internet to develop my digital competence
5.4.4	I am able to identify gaps in digital competence and find solutions for them
5.4.5	I am able to do simple activities through ICTs
